# The BioStudies database—one stop shop for all data supporting a life sciences study

**DOI:** 10.1093/nar/gkx965

**Published:** 2017-10-23

**Authors:** Ugis Sarkans, Mikhail Gostev, Awais Athar, Ehsan Behrangi, Olga Melnichuk, Ahmed Ali, Jasmine Minguet, Juan Camillo Rada, Catherine Snow, Andrew Tikhonov, Alvis Brazma, Johanna McEntyre

**Affiliations:** European Molecular Biology Laboratory, European Bioinformatics Institute, EMBL-EBI, Wellcome Genome Campus, Hinxton CB10 1SD, UK

## Abstract

BioStudies (www.ebi.ac.uk/biostudies) is a new public database that organizes data from biological studies. Typically, but not exclusively, a study is associated with a publication. BioStudies offers a simple way to describe the study structure, and provides flexible data deposition tools and data access interfaces. The actual data can be stored either in BioStudies or remotely, or both. BioStudies imports supplementary data from Europe PMC, and is a resource for authors and publishers for packaging data during the manuscript preparation process. It also can support data management needs of collaborative projects. The growth in multiomics experiments and other multi-faceted approaches to life sciences research mean that studies result in a diversity of data outputs in multiple locations. BioStudies presents a solution to ensuring that all these data and the associated publication(s) can be found coherently in the longer term.

## BACKGROUND

Research in the life sciences is supported by a variety of specialized and structured resources that house an increasingly large volume of biological research data, as witnessed by the contributions to this NAR issue. There is also a parallel increase in data supporting-specific studies in biology that can be found as supplemental data linked to articles, or in several general-purpose repositories such as Dryad (1), Figshare (2) and Zenodo (https://zenodo.org/), which have emerged to help with sharing the ‘unstructured’ data that do not fit into specialized repositories. However, these efforts are not well connected to the specialized life sciences databases, and there is a risk that, over time, different data outputs of biological studies become dispersed and disconnected. The practice of citing the data supporting a study in a rigorous manner from a research paper is still a work in progress (doi: 10.1101/100784), and the wide variety of ways in which this may be done is not only making data discovery difficult but is also hampering efforts to credit scientists for sharing their data.

The BioStudies database aims to address some of these concerns (3). It holds high-level metadata descriptions of biological studies, with links to the underlying data in specialized life science databases at the European Bioinformatics Institute (EBI) and/or databases elsewhere, including the general-purpose repositories mentioned above. When necessary, BioStudies can also host data that have not been already deposited elsewhere. By creating a single record into which all the data pertaining to a study can be linked, BioStudies provides the means to ensure all data elements of the study are linked effectively—a particularly challenging task for multi-omics studies in which data might be published over a period of time in multiple locations. This approach also simplifies the citation of related data in a meaningful way.

As of September 2017, the BioStudies database holds more than a million studies, most of them acquired from Europe PMC. Several projects contributed thousands of datasets (see below); if we measure data file sizes, more than one third comes from projects. The number of studies submitted directly by authors is increasing.

## SCOPE

The purpose of BioStudies is to collate all the data about a study, thus improving data discovery through integration across multiple data resources and the literature in the life sciences. BioStudies therefore welcomes depositions of data supporting a biological study, in particular from studies that also have outputs in structured data resources such as those at the EMBL-EBI. While BioStudies accepts all biological data that do not fit into the structured, specialized resources, it also serves as an envelope for multi-omics studies, with the individual data components residing in archives such as European Nucleotide Archive (ENA) (4), Protein Identification Database (PRIDE) (5) or the functional genomics archive ArrayExpress (6). As ELIXIR (the European Infrastructure for data in the life sciences) formulates recommendations on core data resources (https://www.elixir-europe.org/platforms/data/core-data-resources) and deposition databases (https://www.elixir-europe.org/platforms/data/elixir-deposition-databases), the scope of BioStudies is to support studies that use these resources.

## DATA SUBMISSIONS

There are two ways to submit data to BioStudies: one for individual human depositors and one for bulk data pipelines. First, we provide a web-based submission tool (http://www.ebi.ac.uk/biostudies/submissions). Users can upload data files into their BioStudies home directories, and, via a basic web form, describe their datasets according to the simple BioStudies model, that is, provide a title, basic overall description of the study, links to data deposited elsewhere, and, if desirable, descriptions of those data or the files deposited. The depositor retains complete control over their datasets, and can edit their data—adding files or further descriptions—at a later point. BioStudies keeps version history for each dataset (although this feature is not currently exposed in the web view of a BioStudy).

**Figure 1.**
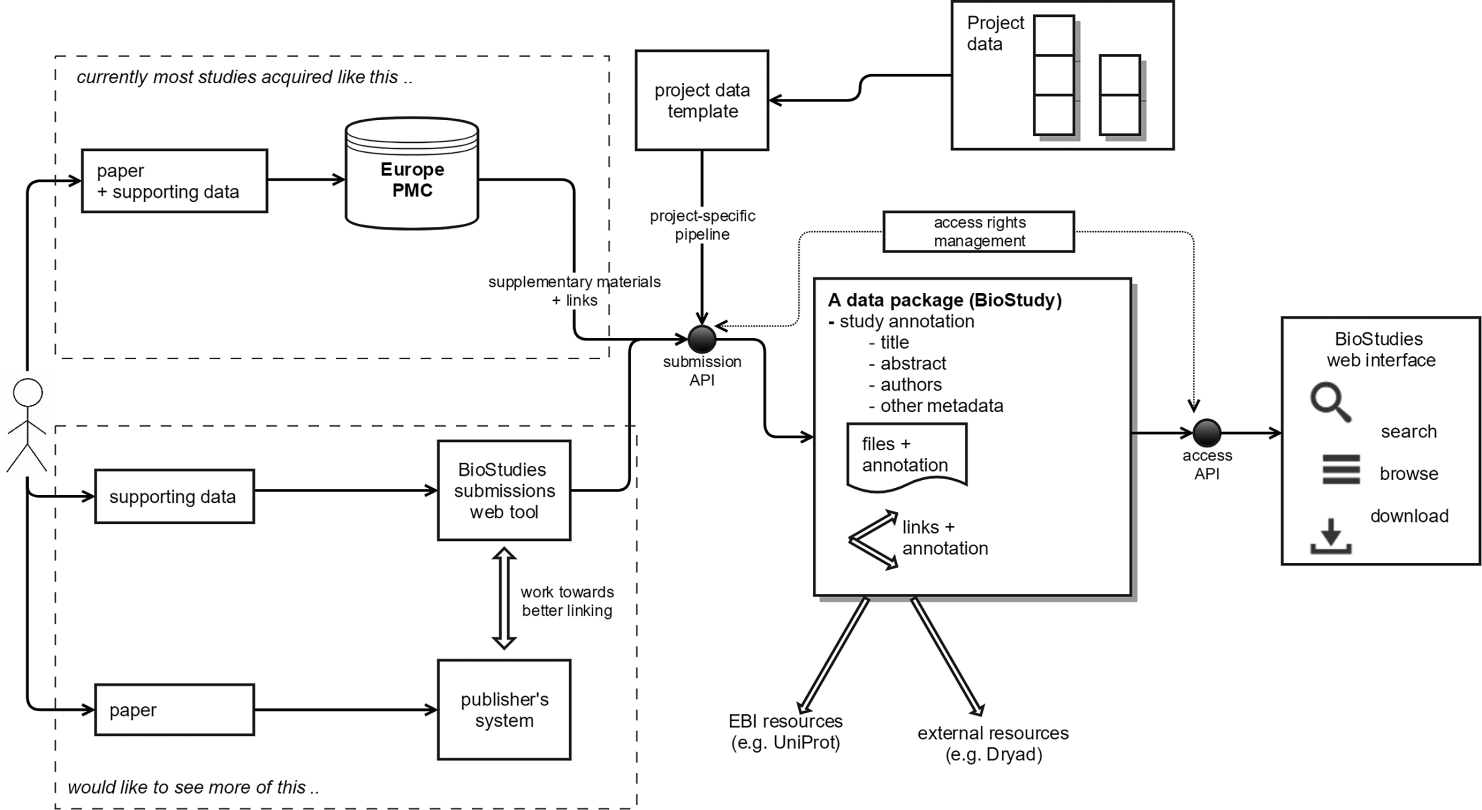
The main concepts of BioStudies, and data submission routes.

The second mechanism is for bulk data submission via a simple tab-delimited file format that can be created in any spreadsheet editing tool such as Microsoft Excel. We call this format ‘PageTab’ (Page layout Tabulation format). It provides means for describing a study, its attributes, the associated files, links, as well as a logical hierarchy for more complex studies. All the information is presented on the website in a manner that closely resembles the input provided. Data is loaded into BioStudies in this format for a number of deposition pipelines, but it can be used also by individual submitters. In Biostudies, a ‘project’ is a group of datasets deposited via a common pipeline; examples of these include Europe PMC, diXa, EurocanPlatform. See Figure 1 for an illustration of data submission routes.

## DATA CONTENTS

To illustrate how BioStudies works in various contexts, here we describe some of the current data sources for BioStudies:

- Europe PMC (7), the database for life sciences literature, provides BioStudies with a data package for all full text papers that contain either supplementary materials, or links to data resources elsewhere, or both. These studies also contain the abstract of the paper, author information, and source of funding extracted from the paper. However, we are encouraging researchers to submit data to BioStudies directly, creating a data package that can be easily referenced from the publication. We will ensure that for papers that have associated data in BioStudies at the time of publication we do not re-import information from Europe PMC. We have started working with various scientific journals to integrate submissions of supplementary material to BioStudies into their pipelines.
- BioStudies serves as a data catalogue for the EurocanPlatform project (8)—see http://www.ebi.ac.uk/biostudies/EurocanPlatform/studies/. This project identified 2710 cancer-related datasets in ArrayExpress and then added a further layer of annotation on them via BioStudies records, which indicate the cancer cell source and the cancer type by linking to the Experimental Factor Ontology—the ontology for describing biological studies and materials at the EBI (9). This demonstrates how BioStudies can be used for managing value-added data for a project, where a dataset for a study already exists in a structured repository (in this case ArrayExpress).
- The diXa data warehouse is a collection of toxicogenomics experimental data, with links to chemical databases (10)—about 300 studies in total. It contains transcriptome, proteome, metabolome and epigenetic data: http://www.ebi.ac.uk/biostudies/diXa/studies/. In this case, we host both the descriptions of the studies, as well as the associated data files.
- Data from several other ongoing projects (http://www.hecatos.eu/, http://www.eu-toxrisk.eu/) are currently held privately, accessible only to project members; these data will be released publicly according to the data management plans of those projects. Capturing project data early increases the likelihood that data will be made public downstream, if it is housed in a resource that can make the private-to-public switch seamlessly, rather that requiring re-upload or re-deposition elsewhere. We are also learning from life sciences communities how to build upstream data capture tools, as well as how to best provide access to data in BioStudies.

Table 1 shows the breakdown of the most popular file types hosted in BioStudies.

**Table 1.** The most popular file formats in BioStudies

File type	Number of files
pdf	667 349
doc, docx	401 381
tif, tiff	294 085
xls, xlsx	216 331
html	156 684
mov, avi	89 677
jpg	50 643
zip	34 757
cif	30 072
cel	27 817

## DATA ACCESS

The main interface (http://www.ebi.ac.uk/biostudies/) allows data browsing and searching both within an individual project and across the entire database. The search is supplemented by auto-completion and ontological expansion. This means that, as users type in their search terms, suggested keywords are displayed, alongside the matching ontology terms and ontological hierarchy. If a user searches for a more generic term (e.g. ‘liver disease’), the search hits also studies where only some sub-terms are present (e.g. ‘autoimmune hepatitis’—see http://www.ebi.ac.uk/biostudies/studies/S-EPMC2485412/?query=%22liver+disease%22+ as an example).

Data files can be downloaded either individually or by selecting a subset. For large studies, users can explore the data files by filtering on a keyword and sorting on one of the associated file annotations (e.g. for http://www.ebi.ac.uk/biostudies/studies/S-DIXA-012/—select only files for source named ‘FP002BI_A12’). All public datasets are available for download via FTP and Aspera protocols. BioStudies also shows similar studies to the study currently displayed. Metadata (in the PageTab format) is available for download in a number of formats: JSON, XML and tab-delimited. An authentication and authorization mechanism allows authors to keep their datasets private until the associated paper is published. This mechanism can also be used in ongoing projects; for example, a single read-only user can be created for access to all the data in a project.

**Figure 2.**
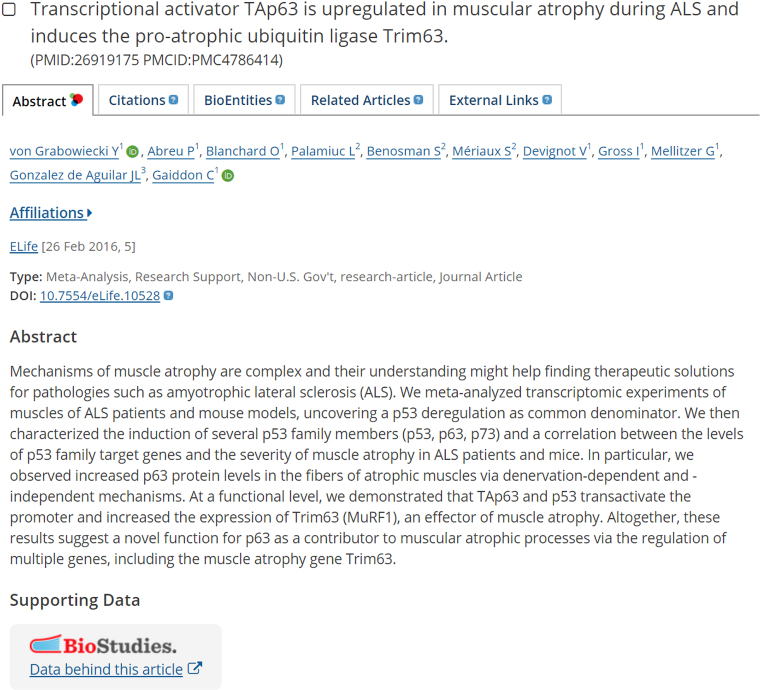
A BioStudies record link from Europe PMC.

BioStudies records are linked from Europe PMC, as appropriate (see Figure 2). All the articles in Europe PMC that have an associated BioStudies record can be found with the following query: http://europepmc.org/search?query=%28LABS_PUBS:%221518%22%29.

## IMPLEMENTATION

The BioStudies infrastructure consists of three largely independent software modules. The BioStudies backend is an application that uses MySQL for data storage and is capable of responding to service requests coming from other components (such as, ‘please log in this user’, or, ‘which projects is this user allowed to submit data into?’). The data access user interface is independent from the backend (except for user authentication) and works on data encoded as JSON, indexed by the Apache Lucene search engine. The data submission tool is written in JavaScript/Angular2, and works on top of its own server side component that takes care of temporary data storage and the communication with the main backend.

## FUTURE PLANS

One of the primary goals of BioStudies is to encourage the emerging practice of citing data in articles by simplifying the process, enabling one reference for all the data associated with a publication. In order to achieve this, we are working with publishers to develop BioStudies into a suitable resource for data that supports any life sciences publication. We are aiming to develop the BioStudies data submission system so that it is easy to link to it from manuscript submission workflows, and will implement data deposition Application Programming Interfaces (APIs) to support such integration.

It is the intention over time that the multiple data resources in a given study will link to BioStudies to make other components discoverable. That is, a record in the European Nucleotide Archive could link to a BioStudy for further information related to that sequence, currently only found as ‘supplemental material’. BioStudies will also be included in the general search mechanism of EBI.

One of the mechanisms for integration between articles, data and researchers across the research lifecycle is persistent identifiers for scientists, ORCIDs (11). We will enable easy linkage of a dataset to a researcher’s ORCID record, both upon the initial submission and post-submission.

EBI is working on the next generation assay data submission tool that will give data submitters a single system for getting data into all EBI resources such as ENA, PRIDE, MetaboLights (12), BioSamples (13). BioStudies will serve an important role in this setup and will capture top-level information for all datasets from a study in a single place. Currently more than 330 000 studies (about one-third) contain links to data in other resources (not including links to Europe PMC, or DOIs); out of those, about 12% studies have links to more than one resource, and it is likely that the importance of supporting distributed data deposition will only increase.

We will continue to work with projects that generate complex data; in particular, the data submission tool will be made configurable via a template mechanism where, depending on a project, it will be possible to specify in advance how a dataset should be annotated.

On the technical side, we will develop and release an API for the data access system, will provide a data filtering and faceting mechanism, and better functionality for dealing with large studies. DOIs can be assigned on request, and we will streamline this procedure. Data submitters will be able to provide a link to their (not yet public) study to enable peer review on embargoed datasets prior to publication. Finally, scalability improvements to all components will enable us to serve large-scale imaging data, in collaboration with the Image Data Resource (IDR) (14).
